# Increased Serum Matrix Metalloproteinase-9 Levels are Associated with Anti-Jo1 but not Anti-MDA5 in Myositis Patients

**DOI:** 10.14336/AD.2018.1120

**Published:** 2019-08-01

**Authors:** Yanjuan Liu, Hui Luo, Li Wang, Caiyan Li, Liyun Liu, Li Huang, Ke Liu, Meidong Liu, Siming Gao, Yizhi Xiao, Honglin Zhu, Xiaoxia Zuo, Quan-Zhen Li, Huali Zhang

**Affiliations:** ^1^Department of Rheumatology, Xiangya Hospital, Central South University, Changsha, Hunan, China; ^2^Department of Pathophysiology, Xiangya School of Medicine, Central South University, Hunan, China; ^3^Department of Immunology and Internal Medicine, University of Texas Southwestern Medical Center, Dallas, TX, USA.; ^4^Sepsis Translational Medicine Key Lab of Hunan Province, Central South University, Hunan, China

**Keywords:** MMP9, Anti-MDA5 antibody, Anti-Jo1 antibody, myositis

## Abstract

Matrix metalloproteinases 9 (MMP9) is a member of the zinc-ion-dependent proteinases family and plays a pathogenic role in chronic inflammatory autoimmune diseases. However, its roles in the pathogenesis of myositis have not been elucidated. In this study, we aimed to determine the gene expression and serum level of MMP9 and their relationship with clinical features and serological parameters in myositis. Our results showed that MMP9 mRNA in peripheral blood mononuclear cells (PBMC) was upregulated in myositis patients compared to that in healthy controls. Myositis patients positive for anti-Jo1 antibodies exhibited significantly higher serum MMP9 than anti-MDA5 positive or antibody-negative patients and healthy controls. However, the presence of interstitial lung disease (ILD) did not affect MMP9 levels. We further identified that anti-Jo1-positive myositis patients showed higher numbers of white blood cells (WBC), lymphocytes and neutrophils; increased levels of creatine kinase (CK), lactate dehydrogenase (LDH), and C-reactive protein (CRP); and higher erythrocyte sedimentation rate (ESR) than anti-MDA5 positive patients. In addition, serum MMP-9 levels were positively correlated with WBCs, neutrophils, CK, CRP, ESR, and LDH in myositis patients. In vitro experiments showed that purified serum IgG from Jo-1-positive patients could stimulate PBMCs to release more MMP9 than the IgG from MDA-5-positive sera. These results indicated that increased MMP9 in anti-Jo1-positive myositis patients was associated with the extent of muscle involvement, but not pulmonary damage. The distinct pattern of serum MMP9 perhaps clarifies the differences in pathophysiology between anti-Jo1 and anti-MDA5 in patients with myositis.

Idiopathic inflammatory myopathy (IIM), also known as myositis, is one of the most common acquired muscle disorders, which can be grouped as follows: dermatomyositis, polymyositis, sporadic inclusion body myositis, and immune-mediated necrotizing myopathy [1]. Although all subtypes are characterized by the loss of muscle fibers and inflammation, each subtype has additional specific immunological, clinical, and morphological features [2].

The matrix metalloproteinases (MMPs) are a family of zinc-ion-dependent proteinases, which are responsible for the selective degradation of different extracellular matrix (ECM) components [3]. MMP9, a member of this family, is a gelatinase expressed by leukocytes, fibroblasts, epithelial, and endothelial cells [4], with substrates that include type IV collagen in the basement membrane and tight junction (TJ) protein [5]. Therefore, it has been implicated in the cellular invasion of the basement membrane. It has been reported that MMP9 activity was increased in aged ischemic muscles of mice [6]. MMP9 also plays a pathogenic role in chronic diseases, such as type one diabetes [7] and chronic inflammatory autoimmune diseases, including rheumatoid arthritis, systemic lupus erythematosus, and systemic sclerosis [8-12]. Its levels were shown to be elevated in sera of patients with systemic sclerosis, which correlated well with the skin involvement degree [12]. Furthermore, increased MMP9 levels in the sera of patients with systemic lupus erythematosus were shown to be associated with neuropsychiatric characteristics, particularly cognitive dysfunction [13]. However, the role of MMP9 in the pathogenesis of myositis has not been completely elucidated.

In the diseased muscle biopsies obtained from patients with polymyositis and dermatomyositis, MMP9 was shown to be overexpressed in the endomysial inflammatory cells and regenerating muscle fibers. Moreover, MMP9 expression was shown to be increased in the muscle membranes of MHC class-I-expressing muscle fibers and auto-invasive CD8^+^ T lymphocytes in the muscle biopsy specimen obtained from a patient with polymyositis. However, no changes in MMP9 levels were detected in the sera of myositis patients [8]. In our previous study, peripheral blood mononuclear cell (PBMC) mRNA transcription profiles were obtained from 24 myositis and 16 controls, showing that *MMP9* mRNA levels were increased 4.63-fold in myositis patients compared with those in healthy controls (unpublished data). Therefore, in this study, we aimed to determine MMP9 gene expression and serum levels in patients with myositis and to explore whether MMP9 may serve as a biomarker for the extent of muscle, skin, and pulmonary damage in these patients.

## MATERIALS AND METHODS

### Patients

We enrolled 148 patients with polymyositis and dermatomyositis admitted to the Department of Rheumatology of Xiangya Hospital, Central South University, China, from November 2013 to June 2015. Fifty-six age- and sex-matched healthy controls were enrolled at the physical examination center in the same hospital. All patients were diagnosed with dermatomyositis or polymyositis based on the criteria of Bohan and Peter or Sontheimer, respectively [14]. Serum samples were obtained from all patients. Of these, anti-Jo1 and anti-MDA5 antibodies were detected in 28 and 52 patients, respectively. The remaining patients were designated as antibody-negative, because the commercial assay, Myositis Profile Euroline (DL1530-1601G, DL1530-1601-3G, DL1530-1601-4G; Euroimmun, Lübeck, Germany), is not available in our clinical laboratory. RNA samples were isolated from 66 patients, including 11 anti-Jo1 positive, 10 anti-MDA5 positive, and 45 antibody-negative patients. Clinical and laboratory data were collected from the medical records at the time of serum sampling. The investigation was approved by the Institutional Review Board of Xiangya Hospital, Central South University, China. Informed consents were obtained from all patients and healthy controls.

### Evaluation of myositis specific autoantibodies

Anti-Jo1 antibodies were detected using a commercial line blot (Euroimmun, Lubeck, Germany). In-house unlabeled protein immunoprecipitation assay with Myc-tagged MDA5 C-terminus overexpressed in HEK293 cells and in-house enzyme-linked immunosorbent assay (ELISA) were used for the detection of anti-MDA5 antibodies. Unlabeled protein immunoprecipitation assays for the detection of anti-MDA5 antibodies were performed in accordance with the following protocols. Briefly, 20 μL of patient serum was incubated with 50 µL of a 50% slurry of Protein G Magnetic Beads (Merck Millipore, Billerica, MA, USA) for 1 h at room temperature. Beads were then incubated with 200 µg whole lysate from human HEK293 cells overexpressing Myc-tagged MDA5 C-terminus (GenBank: AAG34368, 447-1025aa) overnight at 4 °C. Immunoprecipitates obtained by unlabeled IP were separated by 10% SDS-PAGE, transferred to PVDF membrane, and detected with anti-c-Myc antibodies (SC- 40, Santa Cruz Biotechnology, Dallas, TX, USA). The anti-MDA5 antibody was also detected by ELISA. Briefly, 96-well microplates were coated with 0.5 µg/ml recombinant full-length MDA5 antigen (Origene Technologies, MD, USA) overnight at 4°C. Diluted patient serum (1:250) was incubated in the blocked plates for 2 h at 37°C. The plates were then incubated with peroxidase-conjugated anti-human IgG (Abcam Medical and Biological Laboratories). The absorbance was read at 450 nm. The antibody units were calculated from the optimal densities at 450 nm using a standard curve obtained from serial concentrations of serum containing anti-MDA5 antibodies.

### IgG purification

IgG fractions were affinity purified on HiTrap™ Protein G HP (Cat NO. 71-7001-00AR, GE Healthcare, Piscataway, NJ, USA) from 5 ml sera. The final protein concentrations of IgG fractions were evaluated by a quantitative analysis method for total protein. All protocols were consistent with the instructions recommended by the manufacturer.

### Isolation of PBMC and IgG treatment

The heparinized blood was collected, and PBMCs were separated by density gradient centrifugation over Ficoll-Paque PLUS (Cat NO. 17-1440-03, GE Healthcare, Piscataway, NJ, USA) for 20 min at 300 ×*g*. The band containing PBMCs was collected and then transferred to a new 15-ml centrifugation tube. PBMCs were washed twice with PBS. Finally, PBMCs were resuspended at a density of 2.0 × 10^6^ cells/ml in complete medium (RPMI 1640 supplemented with 10% FBS) and treated with 500 µg/ml of purified IgG from healthy control, Jo1-positive, or MDA5-positive sera, respectively, for 24 h.

### Isolation of neutrophils and IgG treatment

The heparinized blood was collected, and PBMCs were separated by density gradient centrifugation over Ficoll-Paque PLUS (GE Healthcare, Piscataway, NJ, USA) for 20 min at 300 × *g*. After the PBMC layer was removed, Ficoll was aspirated carefully and then BD Pharm Lyse^TM^ lysing solution (Cat NO. 555899, BD Biosciences, USA) was added to the remaining layer, which contained neutrophils. After centrifugation at 200 × *g* for 5 min, neutrophils were washed twice with PBS. Finally, neutrophils were resuspended at a density of 2.0 × 10^6^ cells/ml in complete medium (RPMI 1640 supplemented with 10% FBS) and treated with 500 ug/ml of purified IgG from healthy control, Jo1-positive, or MDA5-positive sera for 1 h or 3 hr, respectively.

### Evaluation of pulmonary function and interstitial lung disease (ILD) diagnosis

ILD was diagnosed based on respiratory symptoms such as dyspnea and the presence of typical features including ground-glass opacities, reticulation, or honeycombing on high-resolution computed tomography (HRCT) chest scan, performed by an experienced radiologist [15]. When available, forced vital capacity and lung carbon monoxide transfer factor were used to evaluate the pulmonary function. Of the 148 myositis patients, ILD was diagnosed in 78 patients, while 70 were shown not to have ILD. Twenty-three ILD-positive patients were positive for anti-Jo1 antibodies, while 36 patients with anti-MDA5 antibodies were diagnosed with ILD as well (Table 1).

**Table 1:** Baseline characteristics of all patients enrolled in the study.

Variable	All (148)	Anti-Jo1 Positive (28)	Anti-MDA5 Positive (52)	Antibody Negative (68)
Sex, n (%)				
Female	98 (66.2%)	20 (71.4%)	30 (57.7%)	42 (61.8%)
Male	50 (33.8%)	8 (28.6%)	22 (42.3%)	26 (38.2%)
Diagnosis				
PM	39 (26.3%)	16 (57.1%)	3 (94.2%)	20 (29.4%)
DM	109 (73.6%)	12 (42.9%)	49 (5.8%)	48 (70.6%)
Disease duration	12.4 m (0.5-132 m)	9.9 m 0.5-60 m)	3.8 m (0.5-16 m)	19.6 (0.3-132 m)
Concomitant Medication				
Glucocorticoid	146 (98.6%)	28 (100%)	51 (98.1%)	67 (98.5%)
Immunosuppressive Treatment	118 (79.7%)	17 (60.7%)	42 (80.7%)	59 (86.8%)
IVIG treatment	15 (10.1%)	1 (3.6%)	1 (1.9%)	13 (19.1)
Myositis-associated ILD	72 (50.7%)	23 (82.1%)	36 (69.2%)	13 (19.1%)
Muscle Biopsy	31 (20.9%)	4 (14.3%)	10 (19.2%)	17 (25%)
Treatment Naïve patients	82 (55.4%)	13 (46.4%)	25 (48.1%)	44 (64.8%)

ILD, interstitial lung disease

### Real-time quantitative (q)PCR determination of MMP9 levels in PBMCs

Peripheral blood samples were obtained from myositis patients and healthy volunteers. PBMCs were isolated from the heparinized blood by density gradient centrifugation over Ficoll-Paque PLUS (GE Healthcare, Piscataway, NJ, USA). To determine *MMP9* mRNA level in the PBMCs isolated from the myositis patients and healthy controls, real-time qPCR assay was applied. Primers pairs, targeting *MMP9* and *GAPDH* were used: *MMP9*, forward: 5-GTAGCTGAGGATGCCTTCTCC-3; *MMP9*, reverse: 5-CGCCAACTACGACCGGG-3; *GAPDH*, forward: 5-TGGAAATCCCATCACCATCTT CC-3; *GAPDH*, reverse: 5-GGTTCACACCCATGACG AACA-3. Total RNA was extracted from the PBMCs using TRIzol reagent (Invitrogen Life Technologies, Carlsbad, CA, USA) according to the manufacturer's instructions, and used as a template for complementary (c)DNA synthesis. Afterward, cDNA samples were diluted with distilled water into five volumes and qPCR was performed in 20-μL reactions. Amplification was performed in 40 cycles (30 s at 95°C, 5 s at 95°C, 34 s at 60°C) by ABI7500 system.

### Serum MMP9 level determination by ELISA

MMP9 levels in the sera obtained from patients and controls were determined using a commercial ELISA (DMP900; R&D Systems) in 96-well microplates, according to the manufacturer’s instructions. The microplates were pre-coated with monoclonal anti-MMP9 antibodies. After the addition of samples and standards, an enzyme-linked polyclonal anti-MMP9-antibody was added to the wells, followed by the addition of a substrate solution. The intensity of the developed color, indicating MMP9 levels, was measured at 450 nm.

### Statistical analysis

Statistical analyses were performed using SPSS software (version 19.0). Fisher's exact test and χ^2^ tests were used for the analysis of categorical variables. Continuous variables were compared using the Mann-Whitney test or nonparametric analysis of variance (ANOVA). Spearman’s rank was used to analyze the correlation among white blood cell (WBC) numbers; creatine kinase (CK), lactate dehydrogenase (LDH), C-reactive protein (CRP), and serum MMP9 levels; and erythrocyte sedimentation rate (ESR). *p*-values < 0.05 indicated statistical significance.

**Figure 1.**
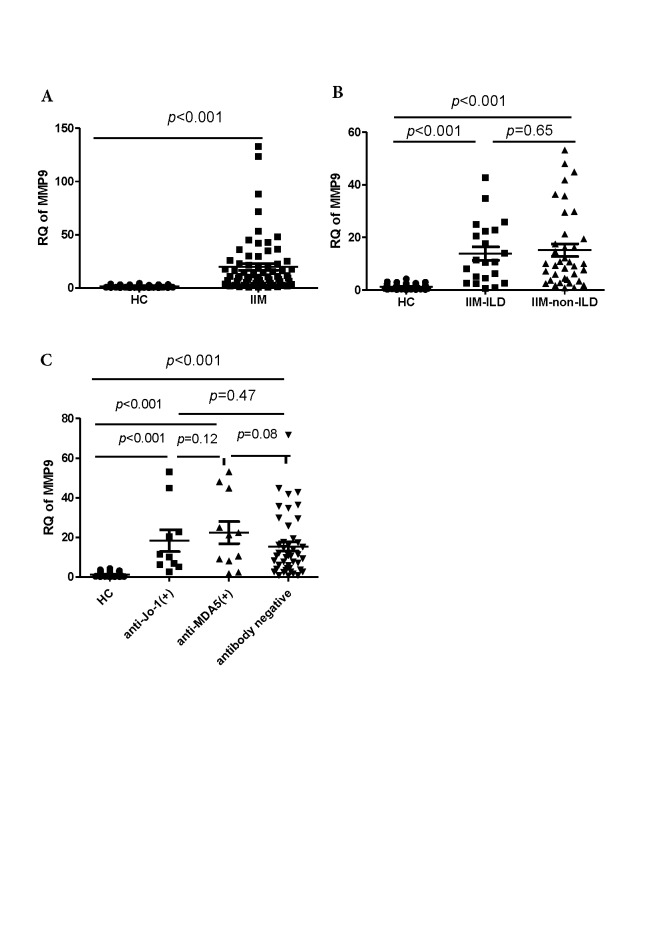
*MMP9* expression levels in cells obtained from patients with myositis and healthy controls (**A**) *MMP9* expression in all patients with myositis and healthy controls. (**B**) *MMP9* expression in healthy controls, and myositis patients with and without interstitial lung disease (ILD). (**C**) *MMP9* in healthy controls, and anti-Jo1 antibody-positive, anti-MDA5 antibody-positive, and antibody-negative patients. HC, healthy control; IIM, idiopathic inflammatory myopathy.

**Figure 2.**
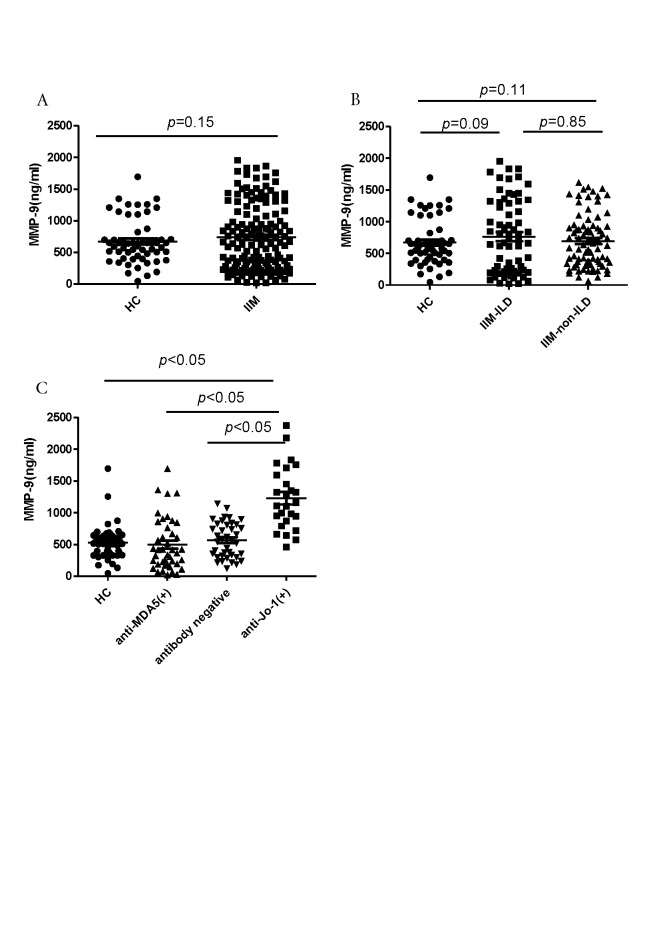
Serum MMP9 concen-trations were determined and compared between different patient groups and controls (**A**) MMP9 levels in healthy controls and patients with myositis. (**B**) MMP9 concentrations in healthy controls and myositis patients with or without interstitial lung disease (ILD). (**C**) MMP9 levels in healthy controls, and anti-Jo1 antibody-positive, anti-MDA5 antibody-positive, and antibody-negative myositis patients.

## RESULTS

### Patient characteristics

A total of 148 patients with myositis were enrolled in our study, including 39 polymyositis and 109 dermato-myositis patients (Table 1). Among these, 66.2% were females. The median age of all myositis patients was 49.5±12.5 years. Some of these patients were diagnosed with ILD as well (50.7%), and the presence of anti-Jo1 and anti-MDA5 antibodies was determined in 18.9% and 35.1% of the analyzed patients, respectively. Of these 148 patients, 118 patients received immunosuppressive agents, including 17 anti-Jo1 positive patients, 42 anti-MDA5 positive patients, and 59 antibody negative patients after hospitalization. The average disease duration of these patients was 12.4 months, ranging from 15 days to 11 years. According to the clinical records, the disease condition of the anti-MDA5 positive patients was more acute and refractory than that of the anti-Jo1 positive and antibody negative patients. In addition, 31 patients were subjected to muscle biopsy, including 4 anti-Jo1 positive patients, 10 anti-MDA5 positive patients, and 17 antibody negative patients. Moreover, 82 treatment naïve patients were enrolled in our study, including 13 anti-Jo1 positive patients, 25 anti-MDA5 positive patients, and 44 antibody negative patients.

### MMP9 gene expression was shown to be increased in patients with myositis

We first determined *MMP9* mRNA levels in PBMCs isolated from 66 patients with myositis and healthy controls; a significant increase in *MMP9* mRNA levels in patients with myositis compared to those in the healthy controls was observed (*p* < 0.001; Fig. 1A). As MMP9 levels were reported to be associated with pulmonary involvement in the progression of autoimmune diseases [16], myositis patients were further sub-classified into the ILD and non-ILD groups, according to the presence of ILD. However, *MMP9* mRNA level did not differ between these groups (*p* = 0.65; Fig. 1B). Anti-Jo1 and anti-MDA5 antibodies are commonly detected in patients with myositis [17]. Therefore, we compared *MMP9* mRNA expression in anti-Jo1 positive patients, anti-MDA5 positive patients, antibody-negative patients, and healthy controls. The results showed that the expression of this gene was significantly upregulated, to a similar degree, in patients with anti-Jo1 and anti-MDA5 antibodies and in antibody-negative patients, compared to that in healthy controls (*p* < 0.001; Fig. 1C).

### Myositis patients with anti-Jo1 antibodies have increased serum MMP9 levels

To determine whether the upregulation of *MMP9* mRNA level is reflected in the serum MMP9 levels as well, we analyzed circulating MMP9 levels in myositis patients and healthy controls. However, these levels did not differ between myositis patients and healthy controls (714.95 ± 517.38 ng/mL *vs*. 527.11 ± 254.07 ng/mL, respectively; *p* = 0.15; Fig. 2A) and between patient groups with and without ILD (828.93 ± 627.83 ng/mL *vs*. 784.49 ± 559.78 ng/mL, respectively; *p* = 0.61; Fig. 2B). However, circulating MMP9 levels were significantly increased in patients with anti-Jo1 antibodies (1127.86 ± 564.40 ng/mL) compared with those in patients with anti-MDA5 antibodies (537.16 ± 454.44 ng/mL), antibody-negative patients (667.88 ± 425.34 ng/mL), and healthy controls (527.11 ± 254.07 ng/mL, *p* < 0.001; Fig. 2C). Moreover, no significant differences in the MMP9 serum levels were found between anti-Jo1 positive ILD patients and anti-Jo1 positive non-ILD myositis patients (*p* = 0.061) or between survival and deceased group (*p* = 0.443).

### Purified IgG from Jo1-positive sera stimulates more MMP9 release by PBMCs than that from MDA5-positive sera

**Figure 3.**
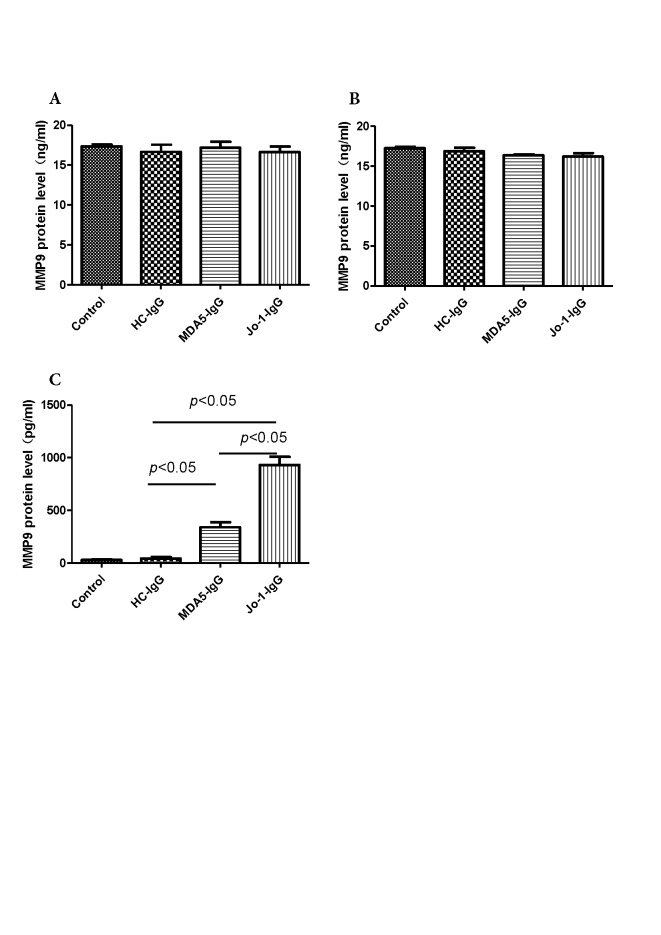
MMP9 concentrations were determined in culture medium of neutrophils or PBMCs stimulated with purified IgG from healthy control, Jo1-positive sera, MDA5-positive sera, or without IgG (**A**) MMP9 levels in culture medium of neutrophils stimulated with purified IgG from healthy control, Jo1-positive sera, MDA5-positive sera, or without IgG for 1 h (n = 4). (**B**) MMP9 levels in culture medium of neutrophils stimulated with purified IgG from healthy control, Jo1-positive sera, MDA5-positive sera, or without IgG for 3 h (n = 4). (**C**) MMP9 levels in culture medium of PBMCs stimulated with purified IgG from healthy control, Jo1-positive sera, MDA5-positive sera, or without IgG for 24 h (n = 4).

To investigate the sources of the increased MMP9 level in Jo-1 positive sera, isolated PBMCs or neutrophils were treated with purified IgG from healthy volunteers, anti-MDA5 positive, or anti-Jo1 positive sera, respectively. Our data showed that there were no significant differences in MMP9 levels among the neutrophil groups treated with purified IgG from healthy control, Jo1-positive, MDA5-positive sera, or without IgG treatment, respectively, for 1 h or 3 h (Fig. 3A and 3B). However, of note, MMP9 protein level was significantly increased in PBMCs treated with purified IgG from both Jo1-positive sera and MDA5-positive sera, to a less extent, compared with that from healthy controls (Fig. 3C). In addition, MMP9 was released much more from isolated neutrophils (17.35 ± 0.44 ng/ml) than from isolated PBMCs (30.08 ± 11.74 pg/ml), suggesting that the major sources of serum MMP9 are neutrophils.

### Treatment status and MMP9 levels

Some articles have reported that glucocorticoids can affect matrix metalloproteinase expression [18]. A relationship between serum MMP9 levels and glucocorticoids was observed in this study. At the time of serum sampling, among myositis patients positive for anti-Jo1 and anti-MDA5 antibodies, 13 patients were treated with glucocorticoids, one patient received immunomodulatory treatment, 16 patients received both glucocorticoids and immunomodulatory treatment, and 38 antibody-positive patients were not treated with any specific medication; we were not aware whether the remaining 12 patients received any specific medication. No significant differences in serum MMP9 concentrations were observed between the groups with or without glucocorticoid treatment (677.49 ± 529.23 ng/mL *vs*. 762.56 ± 602.50 ng/mL, *p* = 0.77)

### Serum MMP9 levels in myositis patients with anti-Jo1 antibodies correlate with some serologic parameters

**Figure 4.**
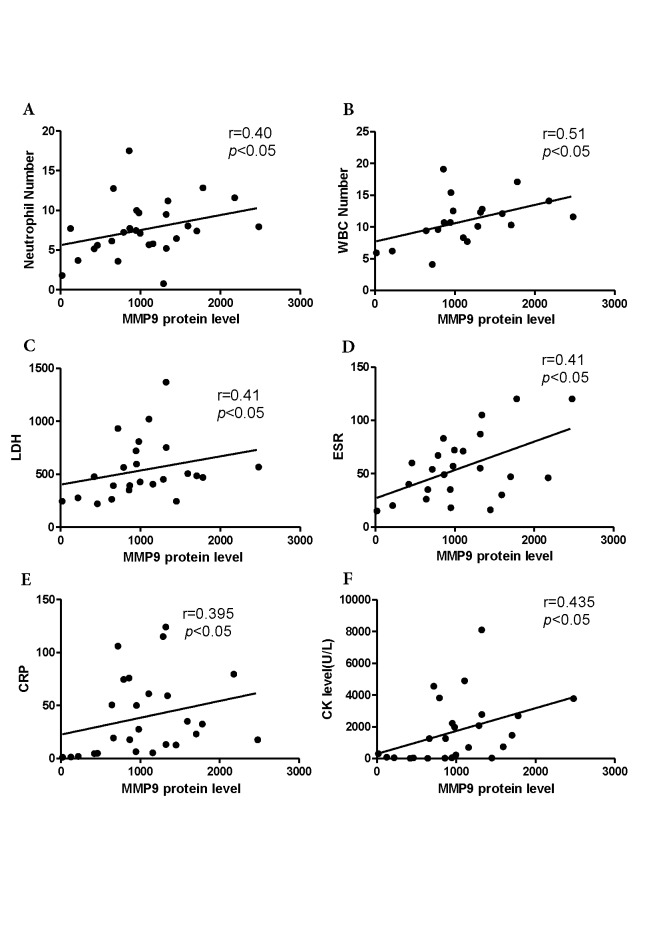
Correlations between serum MMP9 concentrations and serologic parameters in myositis patients with anti-Jo1 antibodies Relationship between MMP9 levels and neutrophil number (A), WBC number (B), LDH (C), ESR (D), CRP (E), or CK (F) in myositis patients with anti-Jo1 antibodies. WBC: white blood cells. LDH: lactate dehydrogenase. ESR: erythrocyte sedimentation rate. CRP: C-reactive protein. CK: creatine kinase.

To investigate the factors contributing to the increased serum MMP9 levels in myositis patients with anti-Jo1 antibodies, serological parameters were compared between anti-Jo1 and anti-MDA5-positive groups (Table 2). WBC (*p* < 0.001), lymphocyte (*p* < 0.001), and neutrophil numbers (*p* < 0.001); CRP (*p* < 0.007), CK (*p* < 0.001), and LDH levels (*p* < 0.006); and ESR (*p* < 0.03) were shown to be significantly increased in the anti-Jo1 antibody-positive myositis groups compared with those in the anti-MDA5 antibody-positive groups. Relationships between serum MMP9 concentrations and serologic parameters in myositis patients with anti-Jo1 antibodies are presented in figure 4. Serum MMP9 concentrations were shown to be positively correlated with WBC (r = 0.51, *p*<0.05) and neutrophil numbers (r = 0.400, *p*<0.05); CK (r = 0.435, *p*< 0.05), CRP (r = 0.395, *p* < 0.05), and LDH levels (r = 0.41, *p*<0.05); and ESR (r = 0.41, *p*< 0.05). Additionally, we did not observe strong correlations between serum MMP9 protein and PBMC mRNA expression levels (r = 0.24, *p* = 0.39).

**Table 2:** Baseline parameter values of 28 patients with myositis with anti-JO1 and 52 patients with anti-MDA5 antibodies.

Parameters	Anti-JO1^*^ patients	Anti-MDA5^*^ patients	*p*
Age (years)	51 ± 10.9	48 ± 10.8	
Temperature (°C)	36.75 ± 0.56	36.70 ± 0.58	0.35
WBCs	11.09 ± 3.62	6.37 ± 3.70	<0.001
Lymphocytes	1.58 ± 0.87	0.882 ± 0.46	<0.001
Neutrophils	5.23 ± 3.09	3.23 ± 1.29	<0.001
Lymphocytes (%)	16.18 ± 8.50	16.34 ± 8.12	0.97
Neutrophils (%)	69.15 ± 18.45	70.58 ± 14.73	0.87
CRP	41.25 ± 37.03	14.76 ± 19.23	0.007
ESR	56.90 ± 32.6	37.70 ± 19.96	0.03
LDH	553.27 ± 284.38	388.62 ± 188.05	0.006
CK	2102.48±2108.27	190.68 ±5 67.94	<0.001
Complement component 3	960.30 ± 207.41	909.70 ± 189.74	0.47
Complement component 4	354.00 ± 223.00	307.46 ± 252.39	0.11

* Data are presented as the mean ± standard deviations; CK, creatine kinase; CRP, C-reactive protein

## DISCUSSION

In this study, we showed that MMP9 mRNA levels in the PBMCs of myositis patients, but not their serum protein levels, are significantly upregulated, which may contribute to the pathogenesis of IIM. Circulating MMP9 levels were shown to be significantly elevated in patients with anti-Jo1 antibodies, but not in those with anti-MDA5 antibodies or antibody negative myositis patients. In addition, MMP9 level in anti-Jo1 positive patients was shown to be significantly correlated with neutrophil numbers; CK, CRP, and LDH levels; and ESR in these patients. The increase in CK and LDH levels in the groups shown to have anti-Jo1 antibodies may indicate muscle involvement [19]. Moreover, no significant differences in serum MMP9 levels were detected between patients with and without ILD or anti-Jo1-positive patients with and without ILD. This is the first report demonstrating that serum MMP9 levels are increased in myositis patients with anti-Jo1 antibodies and that this molecule may be involved in muscle inflammation rather than pulmonary damage or patient survival.

MMP9 can be secreted by peripheral blood cells, such as lymphocytes [20] and neutrophils [21] and it was shown to be stored in granules to be rapidly released after stimulation [22]. Our data demonstrated that MMP9 was secreted by isolated neutrophils without any treatment, and MMP9 was released from isolated PBMCs stimulated with purified IgG from both Jo1-positive sera and MDA5-positive sera, to a less extent. MMP9 released from neutrophils was much higher than that from PBMCs, indicating that the major sources of serum MMP9 are neutrophils. Neutrophil numbers were shown to be significantly increased in the anti-Jo1-positive myositis patients compared with those in the anti-MDA5-positive patients, which is consistent with the higher MMP9 level in anti-Jo1-positive sera than that in anti-MDA5-positive sera. Recently, MMP9 level was found to be induced by CXCL10 from monocytes and neutrophils but not lymphocytes in bullous pemphigoid patients[23], suggesting that the increased MMP9 in PBMCs stimulated with purified IgG from Jo1-positive sera, was perhaps due to monocytes.

MMP9 has emerged as a key factor in the pathogenesis of autoimmune diseases and is involved in small-vessel vasculopathy [13, 24-26]. MMP9 plays an essential role in the migration of immune cells as the presence of lymphocytes, dendritic cells, and neutrophils were reduced in bronchoalveolar lavage fluid when MMP9 was knocked out in mice [27]. Leukocyte-derived MMP9 is also required in EAE (Experimental autoimmune encephalomyelitis) for initial infiltration into the blood-brain barrier[28].

MMP9 level was shown to be increased in the muscle fibers and around auto-invasive CD8^+^ T lymphocytes in patients with polymyositis [29]. Therefore, we hypothesize that, during myositis pathogenesis in patients with anti-Jo1 antibodies, MMP9 is released from neutrophils and PBMCs, cleaving type IV collagen in the basement membranes and allowing CD8^+^ T lymphocyte migration and invasion of the MHC class-I-expressing muscle fibers. Moreover, MMP9 can also cleave myelin compounds, such as type II gelatins and myelin basic protein, leading to the generation of epitopes that induce autoimmunity [30]. A previous study has identified histidyl-tRNA synthetase as the substrate of MMP9, and therefore, it may be able to cleave Jo1 protein and produce new epitopes, inducing the production of anti-Jo1 antibodies [31].

DM and PM are often complicated by interstitial lung disease [32], which is always associated with anti-aminoacyl-tRNA synthetase (ARS), such as anti-Jo-1, or anti-MDA5 antibody [12]. There exist some clinical differences between anti-Jo-1-positive patients and anti-MDA5-positive patients [32-34]. Anti-Jo-1 was associated with a unique clinical subset characterized by myositis, arthritis, mechanic’s hands, Raynaud’s phenomenon, and chronic progressive ILD [35], while anti-MDA5 is always present in clinically amyopathic dermatomyositis (CADM), having typical DM skin rash and increased serum levels of ferritin but little or no muscle involvement [33, 36-39]. Myositis patients positive for anti-MDA5 antibody are always complicated with rapidly progressive ILD, which is resistant to treatment, and with poor prognosis [40]. Gono T compared the cytokine profiles of the anti-MDA5-ILD and anti-ARS-ILD subsets and showed that IL-8 levels were significantly higher, but the ratio of IL-4 to IFN- γ was lower, in anti-MDA5-ILD than in anti-ARS-ILD. Our study demonstrated that serum MMP-9 levels were only significantly elevated in anti-Jo-1 positive patients but not in anti-MDA5 positive patients. The distinct pattern of serum MMP-9 level perhaps clarifies the differences in pathophysiology between anti-Jo-1 positive patients and anti-MDA5 positive patients in myositis.

There are several major limitations of this study, despite the novel and clinically relevant findings. Firstly, the patients recruited into our study differed in the course and severity of their diseases, and they received different treatments. Secondly, the number of anti-Jo1-positive patients in our myositis cohort was relatively small. Thirdly, myositis disease activity assessment tests such as Manual Muscle Test 8 (MMT8), MYOACT, and MITAX were not available for our cohort, making it difficult to associate the disease activity with MMP9 levels. Thus, further studies should be conducted to determine whether the increase in MMP9 was correlated with the severity of muscle involvement in anti-Jo1-positive patients.
